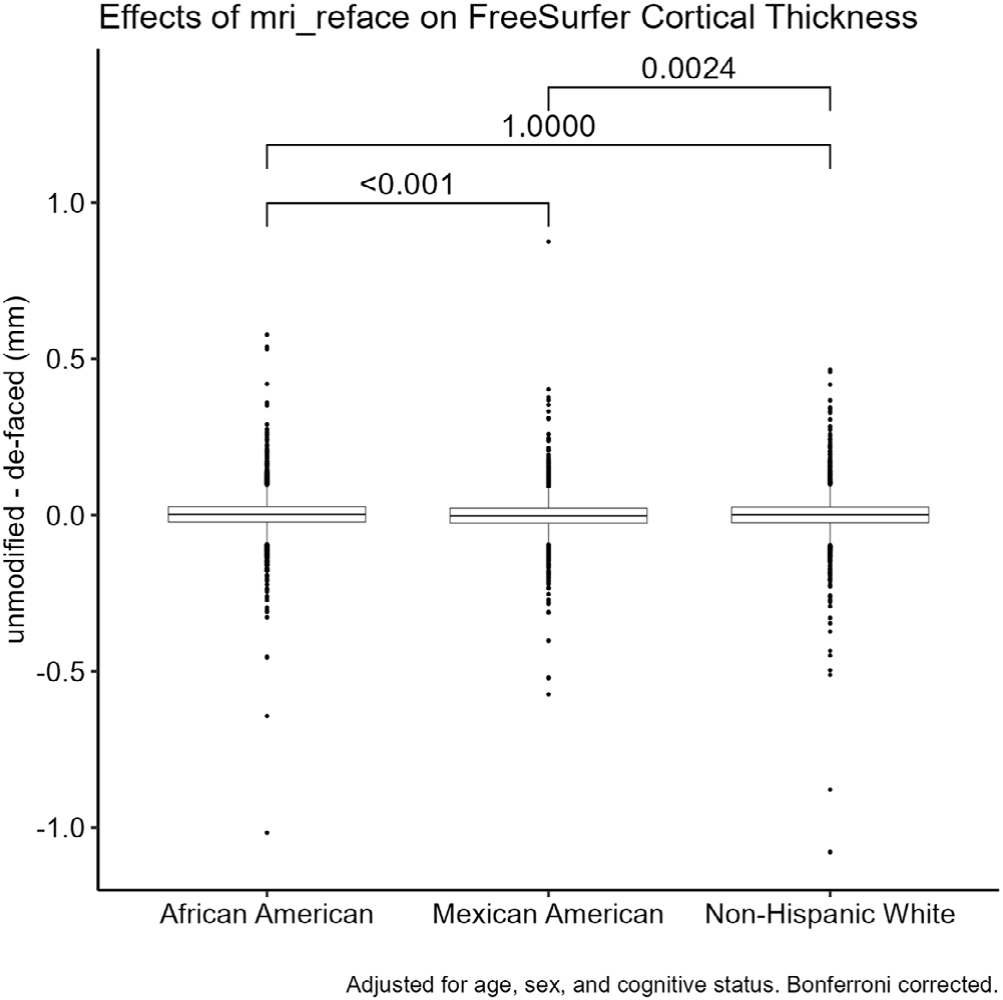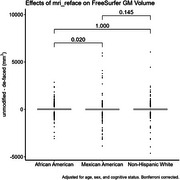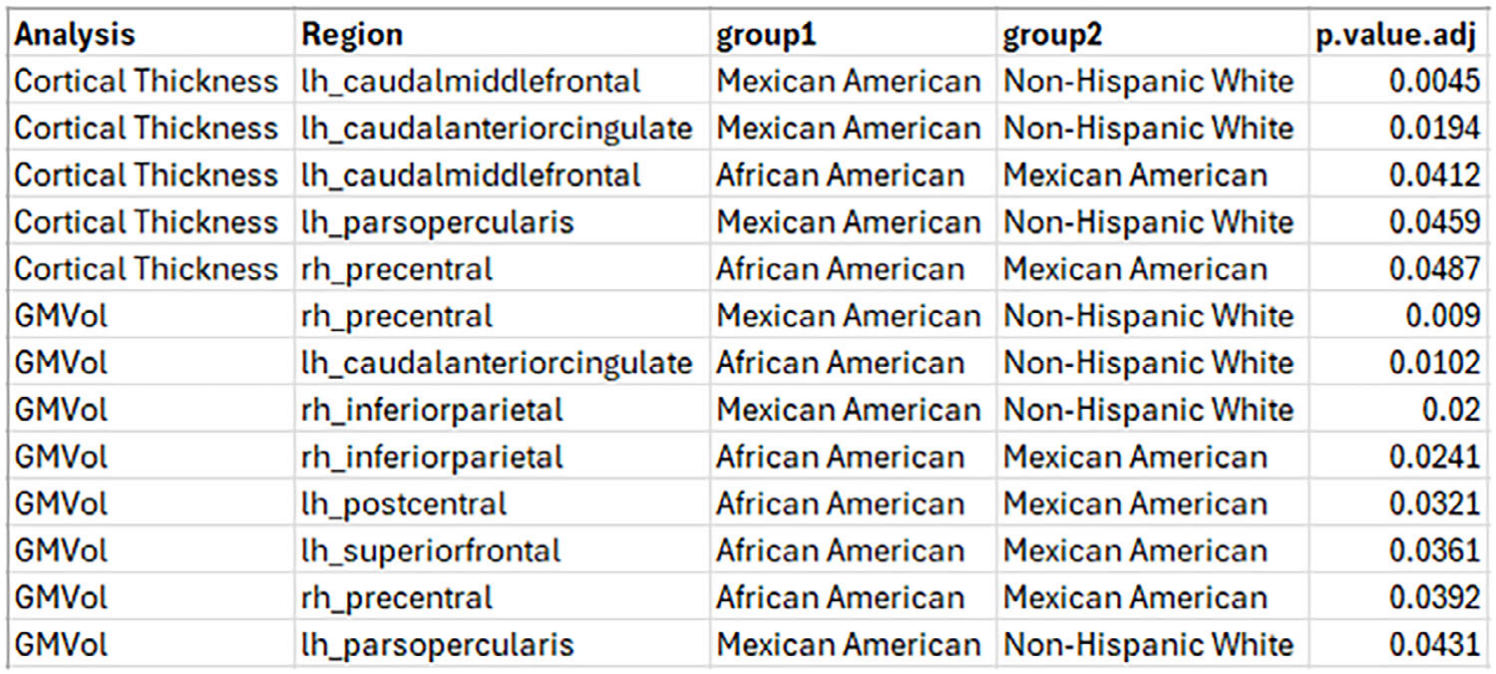# Effects of de‐facing with mri_reface on brain measurements in ethnoracially diverse cohorts

**DOI:** 10.1002/alz70862_109991

**Published:** 2025-12-23

**Authors:** Christopher G Schwarz, Carl M. Prakaashana, Walter K. Kremers, Sid E. O'Bryant, Jeffrey L. Gunter, Matthew L. Senjem, Prashanthi Vemuri, Kejal Kantarci, Jonathan Graff‐Radford, David S. Knopman, Ronald Petersen, Clifford R. Jack

**Affiliations:** ^1^ Mayo Clinic, Rochester, MN USA; ^2^ Institute for Translational Research, University of North Texas Health Science Center, Fort Worth, TX USA; ^3^ Department of Radiology, Mayo Clinic, Rochester, MN USA; ^4^ Department of Neurology, Mayo Clinic, Rochester, MN USA

## Abstract

**Background:**

Use of software to “de‐face” research brain images has grown as advances in automated face recognition have made it increasingly possible to re‐identify participants. Imaging studies of Alzheimer’s Disease and related diseases have included more participants from historically underrepresented groups, but de‐facing software have primarily been validated using images from mostly non‐Hispanic White participants, and its performance has not been directly compared across ethnoracial groups.

**Method:**

From The Health and Aging Brain Study: Health Disparities (HABS‐HD) study, we constructed a sample of 305 T1‐weighted MRI from HABS‐HD baseline visits, matched across three ethnoracial groups (non‐Hispanic White (NHW, *n* = 104), non‐Hispanic African American (AA, *n* = 104), Mexican American (MA, *n* = 97)) according to age‐decade, sex, cognitive status (impaired/unimpaired), and MRI scanner model. We ran *mri_reface* 0.3.5 on each image, and we ran FreeSurfer 7.4.1 on each unmodified image and on each de‐faced output. We then compared regional gray matter (GM) volume and cortical thickness measurements by subtracting each de‐faced image measurement from the corresponding unmodified image measurement. We compared these differences between de‐faced and unmodified images across the three matched groups using GLM analyses adjusted for age, sex, and cognitive status, and Bonferroni corrected.

**Result:**

When aggregating across all brain regions, differences in cortical thickness measures due to de‐facing were significantly different between the NHW and MA groups, and between AA and MA, but not between NHW and AA (Figure 1). Differences between these group means were <0.005mm. For GM volume, differences were significant between AA and MA (magnitude < 0.2%), but not between NHW and AA or NHW and MA (Figure 2). Examining each region individually (Figure 3), cortical thickness differences were significant for only 4 regions (all with magnitudes <0.03mm), and GM volume differences were significant for only 6 regions (all with magnitudes <2%).

**Conclusion:**

Effects of mri_reface on FreeSurfer brain measurements were significantly different across ethnoracial groups. Our future work will optimize mri_reface to eliminate these differences. However, the magnitudes of these differences are sufficiently small that they are unlikely to have a practical effect on analyses of data using current versions.